# X-ray induced damage of *B*_4_*C*-coated bilayer materials under various irradiation conditions

**DOI:** 10.1038/s41598-019-38556-0

**Published:** 2019-02-14

**Authors:** Rolf Follath, Takahisa Koyama, Vladimir Lipp, Nikita Medvedev, Kensuke Tono, Haruhiko Ohashi, Luc Patthey, Makina Yabashi, Beata Ziaja

**Affiliations:** 1 Paul Scherrer Institute, 5232 Villigen PSI, Switzerland; 2 Japan Synchrotron Radiation Research Institute (JASRI), 1-1-1 Kouto, Sayo-cho, Sayo-gun, Hyogo, 679-5198 Japan; 3 RIKEN SPring-8 Center, 1-1-1 Kouto, Sayo-cho, Sayo-gun, Hyogo, 679-5148 Japan; 4 Center for Free-Electron Laser Science CFEL, Deutsches Elektronen-Synchrotron DESY, Notkestrasse 85, 22607 Hamburg, Germany; 5 Institute of Physics, Czech Academy of Sciences, Na Slovance 2, Prague 8, 18221 Czech Republic; 6 Institute of Plasma Physics, Czech Academy of Sciences, Za Slovankou 3, Prague 8, 18200 Czech Republic; 7 Institute of Nuclear Physics, Polish Academy of Sciences, Radzikowskiego 152, 31-342 Kraków, Poland

## Abstract

In this report, we analyse X-ray induced damage of *B*_4_*C*-coated bilayer materials under various irradiation geometries, following the conditions of our experiment performed at the free-electron-laser facility SACLA. We start with the discussion of structural damage in solids and damage threshold doses for the experimental system components: *B*_4_*C*, *SiC*, *Mo* and *Si*. Later, we analyze the irradiation of the experimentally tested coated bilayer systems under two different incidence conditions of a linearly polarized X-ray pulse: (i) grazing incidence, and (ii) normal incidence, in order to compare quantitatively the effect of the pulse incidence on the radiation tolerance of both systems. For that purpose, we propose a simple theoretical model utilizing properties of hard X-ray propagation and absorption in irradiated materials and of the following electron transport. With this model, we overcome the bottleneck problem of large spatial scales, inaccessible for any existing first-principle-based simulation tools due to their computational limitations for large systems. Predictions for damage thresholds obtained with the model agree well with the available experimental data. In particular, they confirm that two coatings tested: 15 nm *B*_4_*C*/20 nm *Mo* on silicon wafer and 15 nm *B*_4_*C*/50 nm *SiC* on silicon wafer can sustain X-ray irradiation at the fluences up to ~10 *μ*J/*μm*^2^, when exposed to linearly polarized 10 keV X-ray pulse at a grazing incidence angle of 3 mrad. Below we present the corresponding theoretical analysis. Potential applications of our approach for design and radiation tolerance tests of multilayer components within X-ray free-electron-laser optics are indicated.

## Introduction

Multilayer mirrors are optical elements used for many applications including on free-electron-lasers (FELs) beamlines. They are composed of multiple thin layers of dielectric material, typically deposited on a bulk substrate. By choice of the type and thickness of the layers, one can design an optical coating with specified reflectivity at different wavelengths of light^1^. Boron carbide (*B*_4_*C*) is frequently used as a reflective coating material for X-ray mirrors both in the soft and in the hard x-ray regime, in particular, due to its high radiation tolerance^2,3^.

Bulk *B*_4_*C* is a semiconducting material of a complex molecular structure, with at least two possible stoichiometries. As X-ray diffraction measurements^4^ demonstrated, it contains a mixture of CBC chains and B_12_ icosahedra, forming complex crystal phases. Correspondingly, *B*_4_*C* properties strongly depend on the local carbon content, i.e., they are inhomogeneous. The details are discussed and the respective phase diagrams are shown in Fig. 2 of ref.^4^. Band structure of *B*_4_*C* includes a band gap of *E*_*gap*_ = 2.09 eV, with a rich substructure which contains electron traps and excitonic levels (see Fig. 13 in^4^).

With the increasing use of free-electron-laser facilities, especially in the hard X-ray regime, where each pulse carries energy of several hundreds of microjoules, there is a quest for methods to increase radiation tolerance of optical elements at FEL beamlines, in particular, of X-ray mirrors. Radiation-hard *B*_4_*C* coating is a promising alternative to other mirror-coating materials, as already investigated, e.g., by A. Aquila *et al*. in^5^. Such coating may also contain an additional layer of a radiation-hard material, with an interface to the supporting Si wafer^2^.

We performed a dedicated experiment on a *B*_4_*C* coated bilayer system at the free-electron-laser facility SACLA. It followed a series of earlier experiments by T. Koyama and SPring-8 Optics group^6–8^. Two coatings were tested: 15 nm *B*_4_*C*/20 nm *Mo* on silicon wafer and 15 nm *B*_4_*C*/50 nm *SiC* on silicon wafer. The system has been irradiated with linearly polarized 10 keV X-rays (at horizontal polarization) at a grazing incidence angle of 3 mrad (0.17°). The experimental setup and measurement geometry were the same as in the earlier experiment described in detail in ref.^6^. Both coatings sustained the irradiation at the fluences up to ~10 *μ*J/*μm*^2^ (=1000 J/cm^2^). Below we perform a corresponding theoretical analysis. The model that we will propose can also be applied for other materials and irradiation conditions.

Firstly, we discuss the structural damage and the theoretical damage threshold doses for *B*_4_*C*, *SiC*, *Mo* and *Si*, and compare them to the available experimental data. Later, we model the irradiation of the experimentally tested coated bilayer systems under two different conditions of an X-ray pulse incidence: (i) grazing incidence, and (ii) normal incidence, and compare quantitatively the effect of the pulse incidence on the radiation tolerance of the bilayer systems. Finally, we present conclusions, and outline prospective model applications.

## Structural Damage

### Microscopic mechanisms of radiation damage in solids

Irradiation with X-rays delivers energy to the material through photoabsorption processes. Photo- and Auger electrons released from the valence band and deeply-lying atomic shells ionize the material further through impact ionizations. As a consequence, further electrons are released which form electron cascades. As it was shown in, e.g.^9–12^, if the radiation dose absorbed is high enough, the number of excited valence electrons exceeds a certain critical value which may trigger a non-thermal structural transition in covalently bonded materials. It occurs due to the presence of many excited electrons which strongly perturb the interatomic potential. Such perturbation forces atoms to move from their initial positions, initiating structural changes within the material. This type of transition is called non-thermal phase transition^9^, as its duration is too short for advancing an energy exchange between hot electronic system and lattice which would lead to a raise of the lattice temperature, typically on ps timescales. Typical timescales for non-thermal transitions are of the order of a few hundred femtoseconds. Therefore, the atomic system still remains cold while a non-thermal transition occurs. Recently, a non-thermal transition has been observed for diamond^13^ on the timescale of 150−200 fs.

The lattice heating due to the electron-lattice energy exchange may also trigger a structural transition. However, as mentioned above, it typically occurs on timescales longer than a non-thermal transition. I.e., if the absorbed dose is higher than the non-thermal transition threshold, both thermal and non-thermal processes may occur. However, in this case, the non-thermal transition switches on earlier and is, therefore, the dominant damage channel. If the absorbed dose is lower than the non-thermal transition threshold, only thermal structural transition may occur. It then also triggers structural changes, typically on a picosecond timescales. The examples of non-thermal and thermal transitions in solids and of their interplay are discussed in refs^14,15^.

Metals - in contrast to semiconductors and dielectrics - predominantly experience thermal transitions^10,16^. The potential-energy surface in metals on which atoms move is only slightly affected by electronic excitation. Thermal mechanism becomes then a predominant channel for material melting. However, if the deposited dose is high enough, the timescale of such thermal transition shrinks, and can even enter a subpicosecond regime as discussed e.g., in^10^ (see also references therein).

### Definition of threshold dose for structural damage

In contrast to irradiation with optical laser pulses, irradiation with X-rays yields to a large degree a uniform volumetric heating of the exposed materials down to a depth corresponding approximately to the penetration depth of photons in the considered material. By the X-ray penetration depth, *d*_*XP*_, we understand here the depth into the material, measured along the surface normal where the intensity of X-rays falls to 1/*e* of its value at the surface - adapting the convention from^17,18^. It is a function of the X-ray incidence angle and photon energy. An universal measure for the damage threshold, *D*_*damage*_, can then be introduced. It is a minimal average radiation dose absorbed per atom at which a structural damage of the material appeared. The absorption ‘per atom’ refers only to atoms located in the material layer down to the photon penetration depth. Under ‘damage’, we understand here any phase transition changing the macroscopic properties of the target. We do not include into this definition such effects as point defect creation, surface roughening etc.

Thermodynamic considerations indicate that structural thermal and non-thermal phase transitions occur at a certain radiation dose absorbed per atom^11^. This critical dose weakly depends on the way the energy is delivered to the atomic system, provided that the energy dissipates slowly out of the system. Therefore, the average dose per atom can conveniently be used to diagnose structural damage under various irradiation conditions, e.g., at various pulse fluences or X-ray photon energies. For example, for diamond the structural damage threshold is: *D*_*damage*_ ~ 0.7 eV/atom^11,19^ for a non-thermal transition. For silicon: *D*_*damage*_ ~ 0.65 eV/atom for a thermal transition, and *D*_*damage*_ ~ 0.9 eV/atom for a non-thermal transition^15^.

The energy absorbed during X-ray irradiation is initially transferred *only* to the electronic system. Therefore, the damage threshold dose can be converted into a critical number of secondary electrons released per atom. Let us introduce a factor *R*, describing the ratio of the excited electrons per atom, *N*_*exc*_ to the number of valence electrons per atom, *N*_*val*_, i.e., *R* = *N*_*exc*_/*N*_*val*_. The critical fraction of excited electrons *R*_*damage*_ can then be related to the damage threshold dose, *D*_*damage*_ as:

1 $${R}_{damage}\simeq {D}_{damage}/({N}_{val}\cdot {E}_{eh}),$$

where *E*_*eh*_ is average electron-hole pair creation energy, 2 · *E*_*gap*_ ≤ *E*_*eh*_ ≤ 3 · *E*_*gap*_, with *E*_*gap*_ being a band gap width^20,21^. Knowing *D*_*damage*_, one can estimate from Eq. (1) the critical fraction of excited electrons for the material of interest. The estimates for *B*_4_*C*, *SiC*, *Si* and for diamond are summarized in Table 1, together with the experimentally measured damage thresholds. They are valid for damage induced by X-ray radiation pulses of the duration < 20 fs, i.e., shorter than the timescale of damage formation. A specific temporal shape of the pulse then does not play a role^12^. An informative compendium on the available measured damage threshold doses, with the references to the original measurements, can be found in refs^2,3^. and, for diamond, in ref.^19^.

**Table 1:** Damage threshold doses estimated from experimental data and the corresponding critical fractions of X-ray excited electrons calculated with Eq. (1) for the constituents of coated bilayer systems (tested here) and for diamond.

Sample	*D*_*damage*_ [eV/atom]	*R*_*damage*_ [%]
*B*_4_ *C*	~0.75	3.7–5.6
*SiC*	~0.94	3.3–4.9
*Si*	~0.90	6.7–10.0
*C*(*diamond*)	~0.70	1.0–1.6

In particular, dedicated microscopic simulations^11,15^ have shown that at the threshold dose for non-thermal damage, the critical fraction of excited electrons, *R*_*damage*_ in diamond is ~1.5%. In silicon, it lies at ~6–9%. These values agree well with the estimates from Eq. (1).

### Reasons for discrepancies between the theory predicted and measured damage thresholds

Various measurements of damage thresholds performed at different X-ray wavelengths and pulse fluences^2,3^ yielded the fluence values at which first surface modifications were observed (e.g.^22,23^). The emergence of the surface changes, detectable, e.g., in Raman spectra or diffraction patterns, can be used for the definition of damage threshold: the pulse fluence is the highest at the material surface, which implies that the dose absorbed per atom is also the highest in the vicinity of the surface. Consequently, any X-ray induced structural changes in a material will manifest at its surface. However, if one converts damage threshold fluence values from various measurements^2,3^ into the absorbed dose (eV/atom), one can observe that the dose values obtained for various X-ray radiation wavelengths differ. This discrepancy seems to be in contradiction with the thermodynamical argument made in the previous chapter that thermal and non-thermal structural phase transitions occur at a certain average absorbed dose per atom, independently on the photon energy^11^. According to it, one would expect that delivery of the same amount of energy per atom within the system (here, even within a similar time interval) should trigger the same transition. This seeming controversy can be resolved by noticing that the measurements at different wavelengths may be affected by the transport of energetic electrons^9^, as well as by the electron emission off the surface. For further details, see the Section “Theoretical model”.

To understand the discrepancy, let us recall that our definition of damage threshold assumes that the radiation energy absorbed by the material in the ‘photointeraction’ volume, i.e., down to the photon attenuation length, *remains* in this volume, i.e., also the photo- and secondary electrons created within the volume do not leave it. In reality, this assumption is fulfilled only if the photon penetration depth is large when compared to the photoelectron range. The ideal measurement of the damage threshold could be performed with hard X-rays arriving at normal incidence angle into the material. For illustration, in boron carbide crystal, the penetration depth of 10 keV photon is ~3000 *μ*m, i.e., very large when compared to the corresponding photoelectron range of ~1.3 *μ*m. Under such conditions, electronic transport out from the photointeraction volume is neglibile. Otherwise, energetic electrons can quickly carry the energy out of the photointeraction region deeper into the material, effectively decreasing the absorbed dose in this region^5,6^.

Therefore, the estimated damage threshold fluences (i.e., the damage threshold doses) from experimental data approach the corresponding theoretical estimates only at short X-ray wavelengths. It is demonstrated, e.g., by the measurements of *B*_4_*C* damage dose presented in^2^ (see Fig. 4 therein) and in^3^ (see Fig. 4 therein). There, the estimated damage thresholds^23,24^ converge to the theoretical ones only at the short X-ray wavelengths. We show those converged values in Table 1.

## Theoretical Model

Below we present a simple theoretical model, capable of estimating damage thresholds in multilayer materials under varying X-ray incidence and polarization. Its predictions will be later verified by a comparison to the experimental data obtained from our SACLA measurement.

Let us emphasize that we do not aim here to construct a high-accuracy quantitative model that would require time-consuming large-scale calculations. Our intention is to offer a versatile tool capable to roughly *estimate* damage thresholds in various materials within multilayer systems.

First, we calculate the density of photoelectrons, *n*_*photoel*_(*x*) produced at various depths within the irradiated system, after the X-ray pulse is over. The number of photoelectrons created by X-ray pulse per unit surface between depths *x* and *x* + *dx* in the material equals to the number of absorbed photons, and, consequently, to the pulse energy absorbed between those layers, divided by the photon energy:

2 $$d{N}_{photoel}(x)=d{F}_{abs}(x)/{E}_{\gamma },$$

where *F*_*abs*_(*x*) denotes the absorbed pulse fluence. Assuming the Beer-Lambert law for X-ray absorption, the pulse fluence at depth *x* is:

3 $$F(x)={F}_{T}\cdot \exp (\,-\,x/{d}_{XP}),$$

where fluence *F*_*T*_ denotes the fluence at the material surface which was transmitted into the material, *F*_*T*_ = *F* · (1 − *R*) · sin *α*, with *F* being the experimental XFEL fluence, estimated from the beam energy divided by the lateral beam focus, and *R* being the reflectivity of the surface at the given grazing incidence angle *α*. Respectively, *dF*_*abs*_(*x*) = *F*(*x*) − *F*(*x* + *dx*), which yields the following expression for the density of photoelectrons, *n*_*photoel*_(*x*) ≡ *dN*_*photoel*_(*x*)/*dx*:

4 $${n}_{photoel}(x)={F}_{T}\cdot \exp (\,-\,x/{d}_{XP})/({E}_{\gamma }\cdot {d}_{XP})$$

Photoelectrons interact further with the sample, creating cascades of secondary electrons. Let us recall that the 3D secondary electron density, *n*_*el*_(*t*, **r**), at some time instant *t* and at spatial position **r,** can be estimated from a convolution of 3D photoelectron density, *n*_*photoel*_(*t*, **r**) and the density of secondary electrons created by one photoelectron, *n*_*el*|1*photoel*_(*t*, **r**):

5 $${n}_{el}(t,{\bf{r}})=\int {d}^{3}{\bf{r}}{\boldsymbol{^{\prime} }}\,\int dt^{\prime} \,{n}_{photoel}(t^{\prime} ,{\bf{r}}{\boldsymbol{^{\prime} }})\,\,{n}_{el|1photoel}(t-t^{\prime} ,{\bf{r}}-{\bf{r}}{\boldsymbol{^{\prime} }})$$

In our model we resolve only one spatial dimension, i.e., the depth into the material, *x*. Further, for the damage threshold estimation it is sufficient to know the total density of secondary electrons after the electron cascading processes finished. After performing the respective time integration in Eq. (5), and reducing the number of spatial dimensions, we arrive at the formula:

6 $${n}_{el}(x)=\int \,dx^{\prime} \,{n}_{photoel}(x^{\prime} )\,\,{n}_{el|1photoel}(x-x^{\prime} )$$

**Figure 1:**
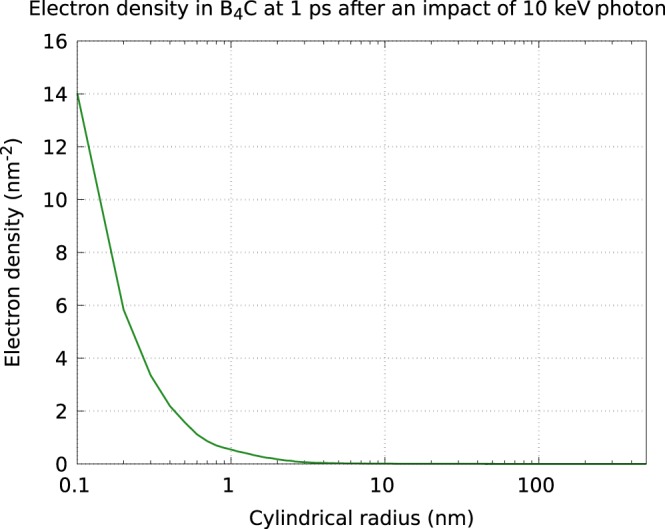
Electron density per surface transversal to the initial velocity of the photoelectron, recorded after the electron cascading was finished.

**Figure 2:**
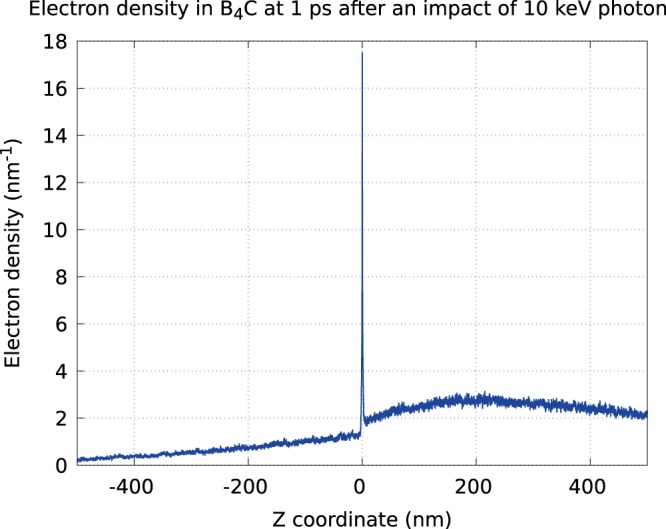
Electron density along the propagation direction of the initial photoelectron, recorded after the electron cascading was finished.

The density of secondary electrons is anisotropic with respect to the propagation direction of the parent photoelectron^25^. Below we demonstrate it on the example of *B*_4_*C* after the impact of a single 10 keV photoelectron (Figs 1 and 2) but this is also true for other materials considered here. The density calculations were performed with XCASCADE (3D) code^26^.

The secondary electron density in the plane transversal to the propagation direction of the parent photoelectron (Fig. 1) is strongly localized and quickly decreases, already at the distance of ~1 nm from the propagation direction.

In contrast, the secondary electron density calculated along the propagation direction of the initial photoelectron (Fig. 2) is asymmetric and, after the initial peak around *z* = 0, it decreases very slowly in the direction of the photoelectron propagation.

In order to correctly include the effect of electron propagation within the irradiated material, the anisotropy should be taken into account. Consequently, the polarization of X-ray beam becomes important, as the photoelectron distribution peaks along the polarization vector (i.e., the direction of the electric field) of the incident light, if it is linearly polarized.

In what follows, for simplicity we restrict to the grazing incidence case and linearly polarized X-ray beam, as in our measurement. If the polarization vector is parallel to the material surface, the density of secondary electrons created by a single photoelectron, *n*_*el*|1*photoel*_(*x*) can be approximated as:

$${n}_{el|1photoel}(x)=G(x,{\sigma }_{transv})\cdot {E}_{\gamma }/{E}_{eh},$$

where *G*(*x*, *σ*_*transv*_) is a normalized Gaussian distribution function centered around 0 with the standard deviation, *σ*_*transv*_ = *d*_*el*,*transv*_/4, and *d*_*el*,*transv*_ is the transversal electron range (e.g., of ~1 nm for *B*_4_*C*). The ratio *E*_γ_/*E*_*eh*_ gives the estimate of the total number of secondary electrons created by an impact of single photon of energy *E*_γ_. As it was discussed previously, we assume that 2 · *E*_*gap*_ ≤ *E*_*eh*_ ≤ 3 · *E*_*gap*_^20,21^, in order to avoid any bias due to a specific choice of phenomenological model for the average pair creation energy.

Similarly, if the polarization vector is perpendicular to the material surface, then *n*_*el*|*1photoel*_(*x*) = G(*x*, *σ*_*longitud*_) · *E*_γ_/*E*_*eh*_, with *σ*_*longitud*_ = *d*_*el,longitud*_/4 and *d*_*el,longitud*_ being the longitudinal electron range (e.g., of ~1280 nm for *B*_4_*C*^27^). For this Gaussian function parametrization, we utilize the fact that photoelectron distribution peaks along the polarization vector in both directions, i.e., it is symmetric with respect to *x*.

The total electron density *n*_*el*_(*x*) can then be obtained after introducing *n*_*el*|*1photoel*_(*x*) into Eq. (6) and performing the integration over *x*′. The effect of possible electron emission from the surface is then taken into account in the calculations through the integration limit at the surface.

Depending on the irradiation condition, in case of multilayer materials, photo- and secondary electrons may cross borders between various material layers. In case of our measurement, the average electron-hole pair creation energies are similar for all materials considered here. This implies that if the secondary electrons would propagate beyond the layer in which their ‘parent’ photoelectron was created, they would contribute to the overall number of electrons created in other layers in the same way as the electrons originating from the photoelectrons created in other layers. Therefore, the border crossings by fast electrons do not lead to an misestimation of the predicted final number of electrons in the layers.

With these observations, we can estimate the total number of (secondary) electrons created in each layer as:

7 $${N}_{el,i}=\int dx\,{n}_{el,i}(x),$$

with i = *B*_4_*C*, *Mo*, *SiC*, *Si*. The integrations are performed between borders of each layer, and for the deepest layer down to the respective electron range in this layer. The ratio of the total number of free electrons created in the ith layer to the total number of valence electrons therein then is: *R*_*i*_ = *N*_*el,i*_/(*N*_*val*_ · *n*_*i*_ · *V*_*i*_). We conservatively assume that the damage threshold of multilayer system is exceeded if in any layer, the interval between the estimated limiting values of *R*_*i*_ starts to overlap with the respective interval of theoretical limiting values, *R*_*damage,i*_.

## Predictions of Our Model for Damage of Coated Bilayer System Irradiated with Hard X-Rays Arriving at Grazing Incidence

**Figure 3:**
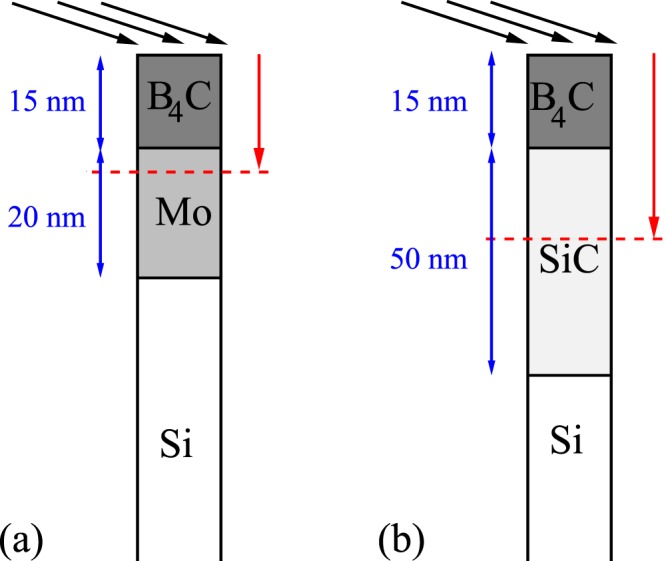
Schematic representation of experimentally tested coated bilayer systems: (**a**) *B*_4_*C*/*Mo* on silicon wafer, and (**b**) *B*_4_*C*/*SiC* on silicon wafer. Black arrows represent X-ray photons, incoming at a grazing incidence into the material. Red arrows indicate the propagation of X-rays into the materials, affecting only the layers close to the surface.

With the model, we studied the damage threshold for our SACLA experiment performed with 10 keV XFEL linearly polarized radiation (horizontal polarization) at the grazing incidence of *α* = 3 mrad. Material parameters of our coated bilayer systems are listed in Table 2. Let us first recall general features of X-ray irradiation at grazing incidence (Fig. 3). At a grazing angle of a few miliradians, the X-ray radiation enters the material surface layer down to several nanometer depth^28^. The reflectivity of the surface is very high, and only a small fraction of the incoming radiation is transmitted into the material. The reflectivities of our samples, with a surface roughness of 0.5 nm, were 0.992^1,17,18^ in both measurement cases. The respective penetration depth is *d*_*XP*_ = 0.0213 *μ*m^17,18^. The grazing angle is close to the critical angle^28^: already at the grazing angle of 4 mrad, the reflectivity decreases to 0.044, with the X-ray penetration depth increasing to 8.2 *μ*m.

**Table 2:** Material parameters of our coated bilayer systems with mass density *ρ*, layer thickness d, and 10 keV photon penetration depth at the grazing incidence angle of 3 mrad.

Sample	*ρ* [g/cm^3^]	d [nm]	*d*_*XP*_ [*μ*m]
*B*_4_ *C*	2.37	15	0.021
*Mo*	10.22	20	0.002
*SiC*	2.85	50	0.006
*Si*	2.33	5·10^6^	0.011

Note that the thickness of the *B*_4_*C* layer is comparable with the respective photon penetration depth, the thickness of the underlying coatings (*Mo*, *SiC*) is ten times larger than the respective photon penetration depth, and the thickness of *Si* wafer is much larger than *d*_*XP*_ for *Si*.

The ‘critical valence electron density’ criterion described above does not apply for metallic molybdenum, for which thermal melting occurs. Thus, we use the thermal damage threshold dose for *Mo*, *D*_*th*_ = 1.19 − 1.26 eV per atom. We estimated it, assuming melting of *Mo* under either constant pressure or constant volume with the respective parametrizations of the latent heat taken from^29,30^.

The model predictions for our experiment at a grazing incidence of 3 mrad are summarized in Table 3. As expected, the irradiation mostly affects the *B*_4_*C* surface layer. In case of the incoming fluence of *F* = 10 *μ*J/*μm*^2^, which is the experimentally estimated damage threshold for both coatings, our model predicts that the surface can still sustain the XFEL radiation. However, the experimental fluence of 13 *μ*J/*μm*^2^ would already damage the outer *B*_4_*C* layer.

**Table 3:** Fractions of excited electrons predicted with our model for the constituents of the coated bilayer systems tested at SACLA.

Layer ith	*B*_4_*C*/*Mo*: *R*_*i*_ [%]	*B*_4_*C*/*SiC*: *R*_*i*_ [%]	*R*_*damage*_ [%]
*B*_4_ *C*	1.9–2.9	1.9–2.9	3.7–5.6
*SiC*	—	0.6–0.9	3.3–4.9
*Si*	1.8·10^−6^ − 2.7·10^−6^	1.4·10^−5^ − 2.2·10^−5^	6.7–10.0
	Dose [eV/atom]	Dose [eV/atom]	*D*_*th*_ [eV/atom]
*Mo*	0.57	—	1.2–1.3

The fractions are compared with the corresponding damage threshold values from Table 1. The incoming fluence is 10 *μ*J/*μm*^2^.

In order to further validate the predictivity of our model, we also calculated the damage thresholds measured in an earlier experiment by A. Aquila *et al*.^5^ under similar irradiation conditions (polarization, grazing incidence). The damage thresholds there were derived for a system consisting of 50 nm *B*_4_*C* layer on a silicon wafer. Together with our predictions, they are summarized in Table 4. Under the given experimental conditions, the penetration depth in *B*_4_*C* is 8.17 nm for 7 keV, 4 mrad, and 5.32 nm for 12 keV, 2 mrad incidence angle, respectively. In both cases the photon penetration depth was ~6–10 times shorter than the thickness of the *B*_4_*C* layer (50 nm).

**Table 4:** Fractions of excited electrons predicted with our model for the constituents of the coated bilayer system: 50 nm *B*_4_*C* on silicon wafer tested by Aquila *et al*. in^5^: case (a) with *E*_γ_ = 7 keV, grazing angle α = 4 mrad, pulse fluence *F* = 24 *μ*J/*μm*^2^, and case (b) with *E*_γ_ = 12 keV, *α* = 2 mrad, and *F* = 100 *μ*J/*μm*^2^.

Layer ith	*E*_γ_ = 7 keV: *R*_*i*_ [%]	*E*_γ_ = 12 keV: *R*_*i*_ [%]	*R*_*damage*_ [%]
*B*_4_ *C*	3.0–4.5	6.2–9.3	3.7–5.6
*Si*	0.001–0.002	0.00005–0.00008	6.7–10.0

The fractions are compared with the corresponding damage threshold values from Table 1.

**Figure 4:**
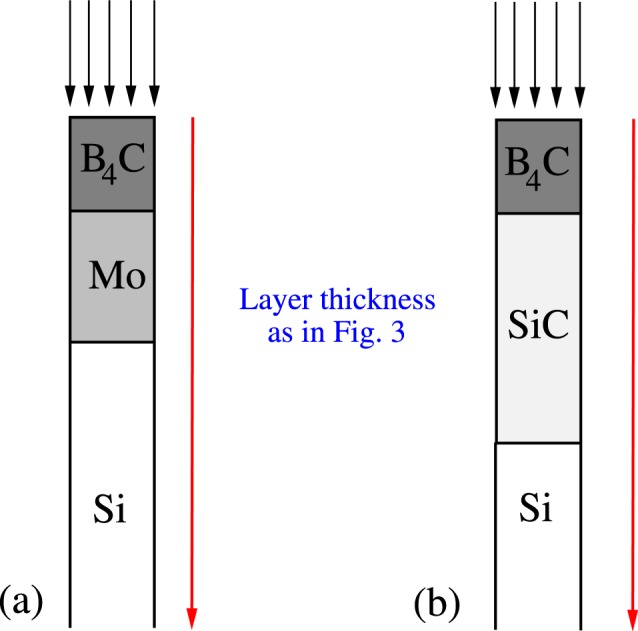
Schematic representation of experimentally tested coated bilayer systems. Black arrows represent X-ray photons, incoming at the normal incidence into the material. Red arrows indicate deep propagation of X-rays into the materials.

According to our model, the estimated damage threshold lies at ~20 *μ*J/*μm*^2^ for 7 keV case. I.e., the irradiated sample is already above the damage threshold at the experimentally estimated damage threshold fluence of *F* = 24 *μ*J/*μm*^2^. One of the reasons for the discrepancy on the theory side could be a different density of *B*_4_*C* layer used in the experiment^5^ that was not explicitly stated in the paper. For our calculation, we used the density value given in Table 2. Additionally, on the experimental side, the fitting of the damage threshold fluence performed in ref.^5^ (Fig. 4 therein) had some finite accuracy which was not specified. For the case with *E*_γ_ = 12 keV, our model predicts a threshold damage fluence of ~40 *μ*J/*μm*^2^ which is lower than the experimentally determined 100 *μ*J/*μm*^2^.

Considering the simplicity of our model and the uncertainty due to the constraints for the electron-hole pair creation energy, the achieved agreement with data is good.

## Predictions of Our Model for Damage within Coated Bilayer System by Hard X-Rays Arriving Under Normal Incidence

To illustrate the strong impact of incidence angle on the damage thresholds in coated bilayer materials, we perform a calculation of the damage thresholds under normal incidence.

X-rays of 10 keV energy arriving at normal incidence onto the material surface can propagate up to a few 1000 *μ*m into the material (Table 5). At such large spatial scales, any first-principle simulation of irradiated multilayer materials (i.e., involving the irradiation processes treated microscopically and including the transport of carriers) would be very time consuming. However, we can still use our model in this regime.

**Table 5:** Photon penetration depth at normal incidence^17^ at the photon impact energy of 10 keV, calculated for the components of our multilayer systems^18^.

Sample	*B*_4_ *C*	*Mo*	*SiC*	*Si*
*d*_*XP*_ [*μ*m]	3156	12	152	134

The damage threshold fluence estimated with the model for the coated bilayer system under normal incidence is ~0.1 *μ*J/*μm*^2^ for the set-up (a) and ~1 *μ*J/*μm*^2^ for the set-up (b) from Fig. 4, i.e., it is ~10–100 times smaller than that one obtained for the case of grazing incidence. The difference is due to the strong absorption of X-rays by *Mo* layer. The respective X-ray penetration depth in *Mo* is 10–100 times smaller than those of the other constituents of our multilayer system (see Table 5).

For comparison with the grazing incidence case, Table 6 shows the electron excitation levels predicted with our model for the considered coated bilayer systems irradiated at normal incidence with a pulse fluence of 10 *μ*J/*μm*^2^ (i.e., equal to the damage threshold fluence at the grazing incidence). Even if the number of electrons in the outer *B*_4_*C* layer stays below the critical value, and the initial (electronic) damage in this layer is suppressed, the damage progressing within the internal layers will also affect thermally the outer layer. Therefore, the *B*_4_*C* layer will be ultimately damaged. Our result then is in agreement with the earlier statement on the detection of damage from the observation of structural modifications at the material surface.

**Table 6:** Fractions of excited electrons predicted with our model for the constituents of the coated bilayer systems tested at SACLA if irradiated under normal incidence with a pulse fluence of 10 *μ*J/*μm*^2^.

Layer ith	*B*_4_*C*/*Mo*: *R*_*i*_ [%]	*B*_4_*C*/*SiC*: *R*_*i*_ [%]	*R*_*damage*_ [%]
*B*_4_ *C*	0.8–1.1	0.8–1.1	3.7–5.6
*SiC*	—	16–25	3.3–4.9
*Si*	43–66	43–66	6.7–10.0
	Dose [eV/atom]	Dose [eV/atom]	*D*_*th*_ [eV/atom]
*Mo*	82	—	1.2–1.3

They are compared with the damage threshold values from Table 1.

To conclude, the material would be destroyed under the experimental fluence irradiation conditions, if the incidence of X-rays were normal.

## Conclusions

In this paper, we proposed a simple model to estimate damage thresholds of X-ray irradiated coated bilayer materials. This versatile tool utilizes specific properties of hard X-ray propagation and absorption in solids, and of the following electron transport. It overcomes the bottleneck problem of large spatial propagation scales, inaccessible for typical first-principle-based simulation tools.

With this model, we analysed structural damage thresholds for complex coated bilayer systems irradiated with linearly polarized X-rays. The predictions obtained for the damage thresholds under grazing incidence were found to be in good agreement with our experimental results, as well as with the earlier results by A. Aquila *et al*.^5^. To demonstrate the strong impact of irradiation conditions on the radiation tolerance of multilayer materials, we also performed a calculation of damage thresholds under normal X-ray incidence with the same pulse parameters as for samples investigated in our grazing incidence experiment. We predicted that the respective damage thresholds are ~10–100 times smaller than those obtained at grazing incidence.

Our model can be used at various grazing angles and for various materials. It can also be extended in a straightforward way to treat multilayer systems and various X-ray polarization schemes. However, if the electron transport between material layers becomes important, the model is only valid if all material layers have similar electron-hole pair creation energy. This is necessary to account correctly for the effect of electron transport between various material layers within the framework of our model. This assumption is fulfilled for the materials used in our study, as well as for many other materials used for construction of X-ray mirrors.

In conclusion, we expect that the model will find a broad range of applications at constructing and testing elements for XFEL optics in the so far computationally inaccessible hard X-ray regime.

## References

[1] Yeh, P. Optical waves in layered media. *J. Wiley and Sons, ISBN: 0-471-73192-7* (2005).

[2] Soufli R (2009). Morphology, microstructure, stress and damage properties of thin film coatings for the LCLS x-ray mirrors. Proc. of SPIE.

[3] Soufli R (2011). Lifetime and damage threshold properties of reflective x-ray coatings for the LCLS free-electron laser. Proc. of SPIE.

[4] Domnich V, Reynaud S, Haber R, Chhowalla M (2011). Boron Carbide: Structure, Properties, and Stability under Stress. J. Am. Ceram. Soc..

[5] Aquila A (2015). Fluence thresholds for grazing incidence hard x-ray mirrors. Appl. Phys. Lett..

[6] Koyama T (2015). Damage to inorganic materials illuminated by focused beam of x-ray free-electron-laser radiation. Proc. of SPIE.

[7] Koyama T (2016). Damage threshold of coating materials on x-ray mirror for x-ray free electron laser. Rev. Sci. Instr..

[8] Kim J (2015). Damage threshold of platinum/carbon multilayers under hard X-ray free-electron laser irradiation. Optics Express.

[9] Sundaram SK, Mazur E (2002). Inducing and probing non-thermal transitions in semiconductors using femtosecond laser pulses. Nat. Mat..

[10] Zier T (2015). Signatures of nonthermal melting. Str. Dyn..

[11] Medvedev N, Jeschke HO, Ziaja B (2013). Nonthermal phase transitions in semiconductors induced by a femtosecond extreme ultraviolet laser pulse. New J. Phys..

[12] Medvedev, N., Tkachenko, V. & Ziaja, B. Modeling of nonthermal solid-to-solid phase transition in diamond irradiated with femtosecond X-ray FEL pulse. *Contrib. Plasma Phys* **55** (2015).

[13] Tavella F (2017). Soft x-ray induced femtosecond solid-to-solid phase transition. H. En. Dens. Phys..

[14] Sciaini G (2009). Electronic acceleration of atomic motions and disordering in bismuth. Nature.

[15] Medvedev N, Li Z, Ziaja B (2015). Thermal and Nonthermal Melting of Silicon under Femtosecond X-Ray Irradiation. Phys. Rev. B.

[16] Recoules V, Clérouin J, Zérah G, Anglade PM, Mazevet S (2006). Effect of Intense Laser Irradiation on the Lattice Stability of Semiconductors and Metals. Physical Review Letters.

[17] Henke B, Gullikson E, Davis J (1993). X-ray interactions: photoabsorption, scattering, transmission, and reflection at E=50-30000 eV, Z=1-92. At. Data and Nucl. Data Tables.

[18] Gullikson, E. X-Ray Interactions With Matter. *Center for X-Ray Optics Database*, http://henke.lbl.gov/opticalconstants (2010).

[19] Gaudin J (2013). Photon energy dependence of graphitization threshold for diamond irradiated with intense XUV FEL pulse. Phys. Rev. B.

[20] Alig R, Bloom S (1975). Electron-Hole-Pair Creation Energies in Semiconductors. Phys. Rev. Lett..

[21] Klein CA (1968). Bandgap Dependence and Related Features of Radiation Ionization Energies in Semiconductor. J. Appl. Phys..

[22] Hau-Riege SP (2007). Damage threshold of inorganic solids under free-electron-laser irradiation at 32.5 nm wavelength. Appl. Phys. Lett..

[23] Hau-Riege SP (2009). Wavelength dependence of the damage threshold of inorganic materials under extreme-ultraviolet free-electron-laser radiation. Appl. Phys. Lett..

[24] Hau-Riege SP (2010). Interaction of short x-ray pulses with low-Z x-ray optics materials at the LCLS free-electron laser. Optics Express.

[25] Ziaja B, London RA, Hajdu J (2005). Unified model of secondary electron cascades in diamond. J. Appl. Phys..

[26] Lipp V, Medvedev N, Ziaja B (2017). Classical Monte-Carlo simulations of x-ray induced electron cascades in various materials. Proc. SPIE.

[27] Berger, M. J., Coursey, J. S., Zucker, M. A. & Chang, J. Stopping-Power Range Tables for Electrons, Protons, and Helium Ions. https://www.nist.gov/pml/stopping-power-range-tableselectrons-protons-and-helium-ions, NIST Standard Reference Database 124 (2017).

[28] Krzywinski J, Cocco D, Moeller S, Ratner D (2015). Damage threshold of platinum coating used for optics for self-seeding of soft x-ray free electron laser. Optics Express.

[29] Choudhury A, Brooks CR (1984). Contributions to the Heat Capacity of Solid Molybdenum in the Range 300–2890 K. Int. J. Thermophys..

[30] Paradis P-F, Ishikawa T, Yoda S (2002). Noncontact Measurements of Thermophysical Properties of Molybdenum at High Temperatures. Int. J. Thermophys..

